# Association Between Fatigue and Falls Risk Among the Elderly Aged Over 75 Years in China: The Chain Mediating Role of Falls Efficacy and Lower Limb Function

**DOI:** 10.3389/fpubh.2022.850533

**Published:** 2022-03-15

**Authors:** Yudi He, Huaguo Zhang, Mi Song, Hongyi Wu, Hongying Pi

**Affiliations:** ^1^Medical School of Chinese PLA, Beijing, China; ^2^Department of Nursing, Beijing Shijitan Hospital, Capital Medical University, Beijing, China; ^3^Department of Nursing, The First Medical Center, Chinese PLA General Hospital, Beijing, China; ^4^Medical Service Training Center, Chinese PLA General Hospital, Beijing, China

**Keywords:** aged, fatigue, accidental falls, lower extremity, self-efficacy

## Abstract

**Background:**

Although fatigue has been shown to be strongly associated with falls risk, very few studies have focused on its mechanism involved in community-dwelling older subjects. The purpose of this study was to explore the relationship between fatigue and falls risk and its internal mechanism by constructing a chain mediation model.

**Methods:**

A cross-sectional study design was adopted. A convenience sample of 270 older adults was recruited from July to October 2021 in an urban community, in Beijing, China. The participants completed the 14-item Fatigue Scale (FS-14), Falls Efficacy Scale International (FES-I), the Short Physical Performance Battery (SPPB) and Fall-Risk Self-Assessment Questionnaire (FRQ) to measure fatigue, falls efficacy, lower limb function and falls risk. The theory of unpleasant symptoms was used as a conceptual framework. Structural equation modeling (SEM) was utilized to test the hypothetical model.

**Results:**

The overall fit of final model was found to be satisfactory: χ*2/df* = 1.61, *CFI* = 0.971, *TLI* = 0.962, *RMSEA* = 0.049 (*95% CI* 0.030/0.066) and *SRMR* = 0.023. Fatigue had a direct effect on falls risk (β = 0.559, *S.E*. = 0.089, *95% CI* 0.380/0.731), and it also had indirect effects on falls risk (β = 0.303, *S.E*. = 0.072, *95% CI* 0.173/0.460) through mediating factors. Falls efficacy and lower limb function were the main mediating variables, and there was a chain mediating effect (β = 0.015, *S.E*. = 0.010, *95% CI* 0.003/0.046).

**Conclusions:**

Our study suggests that fatigue can influence falls risk among the elderly in China. There are many mediating paths between fatigue and falls risk. These results may help healthcare professionals to better understand the inherent relationship between fatigue and fall risk that may benefit older adults.

## Introduction

A fall is defined as an incident in which a person suddenly and involuntarily came to rest upon the ground or surface lower than his/her original station (1). Although falls occur at all ages, older people are more vulnerable to be injured after a fall, creating functionality reduction, loss of independence and, in some cases, death. According to the World Health Organization's estimation, there are nearly 424,000 fatal falls each year (2), which has become a major public health problem all over the world. In China, falls occur in ~13.5% of older adults aged 65 to 74 each year, and the rate increases to 18.3% for those in the 75 to 84 years. For adults aged 85 and older, 22.6% are at risk for falling (3). The occurrence of falls or falls risk depends on extrinsic factors and intrinsic factors. Fatigue has been listed as one main intrinsic factor among older adults (4, 5). Thus, a good way to reduce falls risk is to improve fatigue. However, as often in geriatrics, fatigue is often seen as a marker of aged-related accumulation of deficits (6), making it difficult to intervene against directly. Approaches that indirectly ease the impact of fatigue and falls risk, for instance interventions against mediating factors, may therefore be used instead. Does fatigue affect falls risk through other factors?

The Theory of Unpleasant Symptoms (TOUS), a middle-range theory, is proposed as a means for integrating existing information about a multitude of symptoms. It has three major components: influencing factors (give rise to or affect the nature of the symptom experience), symptoms (the individual is experiencing) and performance outcomes (the consequences of the symptom experience). Influencing factors are those physiologic, psychologic and situational factors that influence the: “occurrence, intensity, timing, distress level and quality of symptoms” (7). The TOUS is one mechanism for providing a diverse and holistic way to understand symptoms experience, including understanding the multidimensional nature of symptoms, as well as the factors influencing symptoms and the consequences of symptoms (8). Therefore, TOUS was chosen as the conceptual framework of our study. In the present study, fatigue was treated as symptoms, falls risk as performance outcomes, then lower limb function and falls efficacy as influencing factors.

Firstly, we hypothesized that lower limb function may be an important mediator variable. For one thing, fatigue may have an impact on lower limb function. A large body of evidences suggested that fatigue older adults experienced was significantly related to the decline of lower limb function, including walking speed, lower mobility and weaker strength (9, 10). Further, participants reporting frequent fatigue at early old age had slower chair rise (β −3.10 rep./min) and TUG speed (β −4.95 cm/s) when compared with those without fatigue (11). For another, lower limb function may have an impact on falls risk. The previous findings confirmed that lower limb strength and gait patterns differed between subjects with and without falls risk (12, 13). Another former study provided insight into the association between balance function and falls risk, concluding that there was an increasing trend across age group in terms of the prevalence of falls and perceived instability among older adults (*X*^2^ = 20.145 and *P*-value < 0.001) (14). In conclusion, both fatigue and lower limb function can affect falls risk, while fatigue can predict lower limb function. Therefore, lower limb function may play an important intermediary role between fatigue and falls risk.

Secondly, we hypothesized that falls efficacy might be another important mediator. Falls efficacy measures older person's perceptions of his or her capabilities in not falling when performing some activities in different environments (15). Research on fatigue and falls efficacy showed that fatigue was significantly associated with falls efficacy (*P*-value ≤ 0.002) (16). Fatigued individuals reported significantly higher falls efficacy scale (FES) (*P*-value < 0.05), indicating less confidence in the performance of daily activities without falling (17). In terms of the relationship between falls efficacy and falls risk, researchers found that falls efficacy was observed to be a predictor of falls risk (18). Furthermore, lower falls efficacy was associated with higher falls incidence (19). The average FES score was significantly higher in fallers (19.2 ± 13.1 for fallers, 14.2 ± 8.3 for Non-fallers, *P*-value = 0.001) (20). All above mean that fatigue can affect an individual's falls risk by improving falls efficacy. Therefore, falls efficacy may also be an important mediator between fatigue and falls risk.

Thirdly, although the above analysis shows that lower limb function and falls efficacy may mediate the relationship between fatigue and falls risk among the elderly, this study holds that they do not simply play an independent mediating role but may also have a chain mediation effect. In addition to the direct effect of lower limb function and falls efficacy on the relationship between fatigue and falls risk mentioned, there is also a correlation between lower limb function and falls efficacy. As people age, the perceived loss of balance in static as well as dynamic situations and decreased muscle strength in daily life may induce lower falls efficacy (21, 22). On multivariate analyses, falls efficacy was independently associated with the Time Up and Go Test (TUG) (*OR* = 1.080, *95% CI* 1.034–1.128) and 4-Stage Balance Test score (*OR* = 0.746, *95% CI* 0.597–0.931), implying the relationship between the quality of gait and balance and falls efficacy (23). Therefore, we speculate that lower limb function can influence falls risk through falls efficacy.

**Figure 1:**
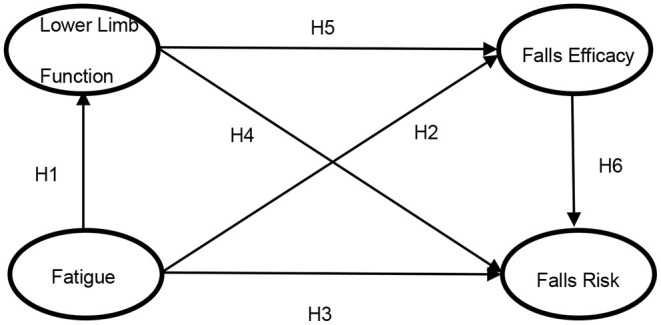
Hypothesized Structural Equation Modeling (SEM).

To our knowledge, very few studies have assessed the link between fatigue and falls risk in community-dwelling older subjects. Will fatigue have an impact on falls risk through lower limb function and falls efficacy? There is a lack of in-depth research on the relationship of falls risk on fatigue through *lower limb function and falls efficacy*, especially the existence of the chain mediating mechanism of falls risk on fatigue through “*lower limb function and falls efficacy*”. Based on these, we plan to construct a chain mediation model in order to explore the relationship between fatigue and falls risk and its internal mechanism this study. Our conceptual model illustrating the hypothesized relationships is presented in Figure 1.

Accordingly, we hypothesized that as:

**H1**. Fatigue will be statistically significant associated with lower limb function of the older adults.

**H2**. Fatigue will be statistically significant associated with falls efficacy of the older adults.

**H3**. Fatigue will be statistically significant associated with falls risk of the older adults.

**H4**. Lower limb function will be statistically significant associated with falls risk of the older adults.

**H5**. Lower limb function will be statistically significant associated with falls efficacy of the older adults.

**H6**. Falls efficacy will be statistically significant associated with falls risk of the older adults.

## Materials and Methods

### Design and Participants

Analysis was based on a cross-sectional study, which was conducted from July to October 2021 in an urban community, in Beijing, China. A convenience sample of older adults were selected to complete this face-to-face survey. Inclusion criteria were as follows: (a) 75 years of age or older, (b) ability to walk without or with a walking aid, (c) stable vital signs, (d) ability to communicate and understand effectively and (e) informed consent to participate voluntarily. The participants were excluded if they had been diagnosed with mental illness, dementia, reading or communication disabilities, or other forms of major disease. All participants could opt out of the survey at any time.

The balance between sample size and overall fit of structural equation modeling (SEM) was quite difficult. According to the research of Schumacker and Lomax, the sample size of most SEM studies is between 200 and 500 (24). Loehlin believed that in SEM model analysis, if the sample size did not reach more than 200, there should be at least 100 samples (25). Similarly, Mueller thought the sample size should be at least 100, and 200 was better (26). Therefore, the sample size of this study was at least 200.

The study was reported following the guidelines of Strengthening the Reporting of Observational Studies in Epidemiology (STROBE). Ethical approval for this survey was obtained. This study also has been supported by a funding.

### Instruments

The 14-item Fatigue Scale (FS-14) (27) is a self-rating scale containing 14 items. It is developed to reflect the severity of two different kinds of fatigue (physical fatigue and mental fatigue). The participants respond yes/no (yes = 1, no = 0) for each item, and the total scales range from 0 to 14 points. Higher scores indicate more severe fatigue symptoms. Cronbach's α ranged from 0.88 to 0.90 for all items.

Falls Efficacy Scale International (FES-I) (28), a 16-item instrument, is consisted of both easy and more complex physical and social activities. It assesses level of concern about falling when carrying out each activity on a four-point scale (1 = not at all concerned, 4 = very concerned). Higher total scores indicate more fear of falling. FES-I is suitable for use in a range of languages and cultural contexts, permitting direct comparison between studies and populations in different countries and settings. Cronbach's α varied from 0.90, 0.921 and 0.97 in German, Chinese and UK samples (29, 30).

The Short Physical Performance Battery (SPPB) (31) measures three components of lower limb function: standing balance, walking speed and chair stands. In the standing balance test, participants attempted to maintain three positions for 10 s each one after the other: side-by-side, semi-tandem and full tandem. Performances were scored as follows: 0: not attempted or tried but unable; 1: the side-by-side but not the semi-tandem for 10 s; 2: the semi-tandem for 10 s but the full tandem for <3 s; 3: the full tandem for 3 to 9 s; 4: the full tandem for 10 s. In the walking speed test, participants were required to walk an 8-foot walking course twice and time on the faster walk was recorded. Quartiles of time were scored as follows: 0: not complete; 1: ≥5.7 s; 2: 4.1 to 5.6 s; 3: 3.2 to 4.0 s; 4: ≤3.1 s. In the chair stands test, participants were asked to rise from a chair and sit down five times as quickly and safely as possible with their arms folded. Quartiles of time were scored as follows: 0: not complete; 1: ≥16.7 s; 2: 16.6 to 13.7 s; 3:13.6 to 11.2 s; 4: ≤11.1 s. The summary performance score ranging from 0 to 12 was created by summing each of the three tests. Cronbach's α was 0.76 for the total scale.

Fall-Risk Self-Assessment Questionnaire (FRQ) is a short, 12-item instrument specifically designed to assess their own fall risks for community dwelling seniors. It was revised by Rubenstein et al. (32) from Vivrette et al. (33). Its items require a yes/no response. The total score can be obtained by summing the total number of points for all “yes” responses (giving 2 points to questions 1 and 5 and 1 point to all other questions). Participants scoring ≥4 (maximum score of 14 with a minimum of 0) are considered to be at an indicated risk. The scale has been applied to Chinese older adults with good internal consistency, with Cronbach's α of 0.724 (34).

### Data Collection

The participants were contacted and recruited through the staff of community health service station. The older adults who met the inclusion criteria were invited to participate in the investigation. Data were collected using structured questionnaires above. A custom-designed questionnaire was used to collect demographic data, including age, sex, ethnicity, marital status, education level, monthly income, financial burden, current dwelling status and current housing type. Due to the age of the participants, the survey was conducted in the form of face-to-face between the investigator and the respondent. To control the quality of survey, all researchers received uniform training before. After completion, the researcher checked whether the items were filled in completely. If there were any missing items, the researcher asked the participant to complete them immediately. The researcher thanked each participant and gave him or her a small gift as a reward after data collection.

### Data Analyses

Descriptive statistics were used to summarize sample characteristics and variables. Correlations between the variables were assessed using Pearson's correlation coefficients. To ensure scientificity and validity of sample data, Harman's single-factor test was conducted to rule out possible common method variance bias (35). All the above statistical methods were completed using IBM SPSS Statistics version 26.0. Structural equation modeling (SEM) was used to test the overall model fit because it is based on a confirmatory approach, allows an examination of the relationships among the observed and latent variables in the hypothesized model and accounts for measurement error (Mplus version 7.4). Maximum likelihood (ML) was used for parameter estimation. Results of effects of variables for the structural model were presented with Estimate and S.E., as well as 95% Confidence Intervals (95% *CI*). Bootstrapping, a nonparametric resampling procedure, is an additional method advocated for testing mediation that does not impose the assumption of normality of the sampling distribution (36). The bootstrap test was sampled for 1,000 times in this study. The goodness of model fit was judged using several statistics including χ*2/df* of 3 or less, root mean squared error of approximation (RMSEA) of 0.08 or less (37), standardized root mean squared residual (SRMR) of 0.08 or less (38), Comparative Fit Index (*CFI*) of 0.9 or more and Tucker-Lewis Index (*TLI*) of 0.9 or more. For all analyses, *P*-value < 0.05 was considered statistically significant.

## Results

### General Characteristics of the Study Sample

**Figure 2:**
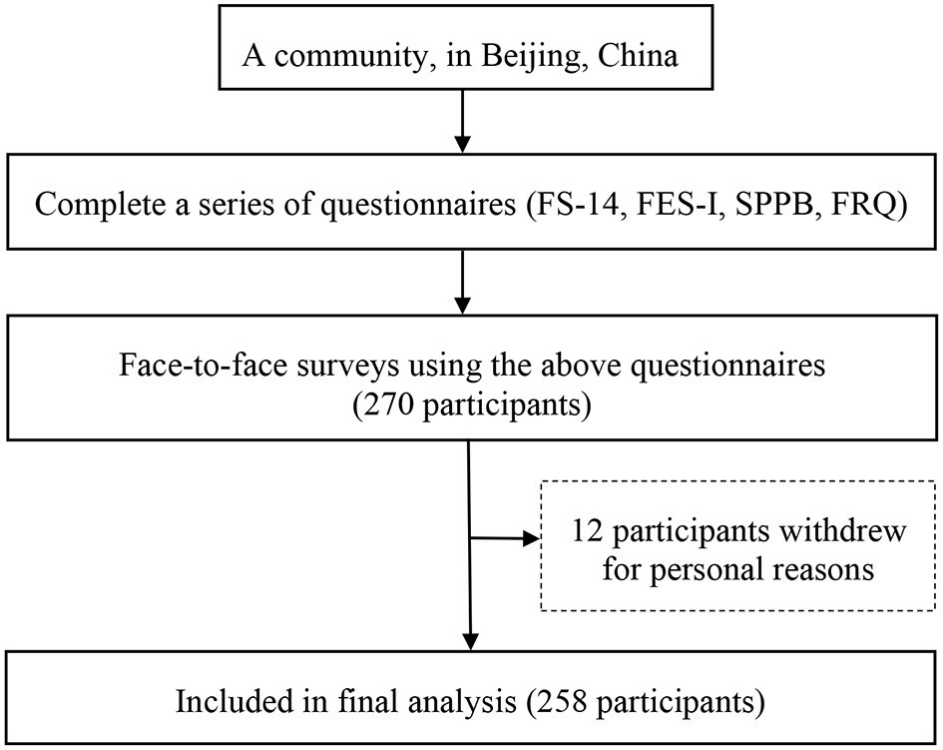
Flow chart of survey process.

A total of 270 participants met the inclusion criteria in this survey, and 12 participants withdrew for personal reasons during the study (Figure 2). Thus, 258 participants (106 males and 152 females) were analyzed, corresponding to a response rate of 95.6%. Their age ranged from 75 to 100 (Mean = 82.72, SD = 5.81). The 12 participants (4 males and 8 females) who withdrew ranged in age from 75 to 86 (Mean = 81.17, SD = 3.81). There is no difference between the sociodemographic characteristics of Non-responders and responders. Table 1 shows the demographic characteristics of the study sample in detail.

**Table 1:** Demographics of the study sample.

**Variables**	**Categories**	**Respondents (*N* = 258)**	**Non-respondents (*N* = 12)**	***P*-value**
Age (years)	75 84	163 (63.2)	9 (75.0)	0.486
	85 94	88 (34.1)	3 (25.0)	
	≥95	7 (2.7)	0	
Sex	Male	106 (41.1)	4 (33.3)	0.594
	Female	152 (58.9)	8 (66.7)	
Ethnicity	Han Chinese	241 (93.4)	12 (100.0)	0.359
	Minority	17 (6.6)	0	
Marital status	Married	217 (84.1)	9 (75.0)	0.458
	Unmarried, divorced or widowed	41 (15.9)	3 (25.0)	
Education level	Elementary school or less	15 (5.9)	1 (8.3)	0.377
	Middle school	39 (15.1)	0	
	High school	62 (24.0)	3 (25.0)	
	University and above	142 (55.0)	8 (66.7)	
Monthly income (RMB)	Up to 1,000	13 (5.0)	1 (8.3)	0.386
	1,001–3,000	32 (12.4)	1 (8.3)	
	3,001–5,000	65 (25.2)	5 (41.7)	
	At least 5,000	148 (57.4)	5 (41.7)	
Financial burden	No burden	144 (55.8)	6 (50.0)	0.915
	Mild	68 (26.4)	5 (41.7)	
	Moderate	33 (12.8)	0	
	Heavy	13 (5.0)	1 (8.3)	
Current dwelling status	Living alone	27 (10.5)	3 (25.0)	0.322
	Not living alone	231 (89.5)	9 (75.0)	
Current housing type	Bungalow	9 (3.5)	0	0.807
	Walk-up apartment	83 (32.2)	4 (33.3)	
	Elevator apartment	166 (64.3)	8 (66.7)	

*RMB, Renminbi*.

### Descriptive Statistics and Correlations of Variables

Harman's single-factor test showed that the maximum unrotated factor in this study could only explain 24.18% of the total variance (<40%), which indicated that the potential common method bias in the sample data was within an acceptable range. The correlations were analyzed to test for multicollinearity among the variables before hypothesis testing. Table 2 presents the descriptive statistics and correlations of the variables. Specifically, a positive correlation was found between fatigue, falls efficacy and falls risk. Low limb function was negatively correlated with fatigue, falls efficacy and falls risk. The correlation coefficients of all the measurement variables in this study ranged from −0.399 to 0.575. Generally, when the absolute value of the correlation coefficient is <0.8, multicollinearity is not considered a problem.

**Table 2:** Descriptive statistics and correlation coefficients among variables in respondents (*N* = 258).

	**Scale range**	**Mean (SD)**	**1**	**2**	**3**	**4**
1 Fatigue	0–14	7.64 (3.21)	1			
2 Lower Limb Function	0–12	9.13 (2.65)	−0.246^**^	1		
3 Falls Efficacy	16–64	23.20 (7.52)	0.483^**^	−0.362^**^	1	
4 Falls Risk	0–14	5.23 (3.04)	0.563^**^	−0.399^**^	0.575^**^	1

** *Correlation is significant at the 0.01 level (two-tailed)*.

### Hypothesized Model Tests

**Figure 3:**
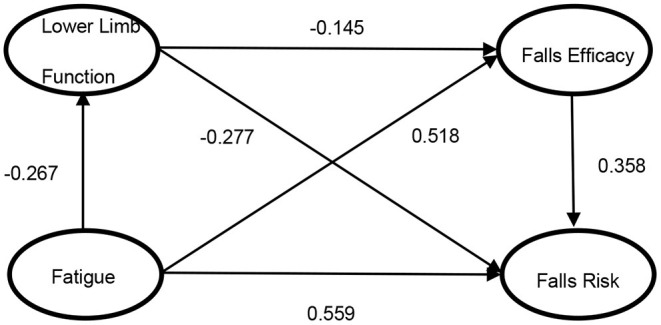
Graphical representations of Final Structural Equation Modeling (SEM).

Figure 3 presents the final model with standardized path estimates. The overall fit of final model was found to be satisfactory: χ*2/df* = 1.61, *CFI* = 0.971, *TLI* = 0.962, *RMSEA* = 0.049 (*95% CI* 0.030/0.066) and *SRMR* = 0.023. Table 3 shows the decomposition of direct and indirect effects of each factor in the structural model. Through bootstrap test, all of our 6 hypothesized paths were supported. There were one direct path (H3) and three statistically significant indirect paths (H1 to H4, H2 to H6 and H1 to H5 to H6). The effect of fatigue on falls risk was divided into direct effect (β = 0.559, *S.E*. = 0.089, *95% CI* 0.380/0.731) and total indirect effect (β = 0.303, *S.E*. = 0.072, *95% CI* 0.173/0.460). Proportion analysis showed that the indirect effect through falls efficacy was largest (67.8%), followed by lower limb function (27.1%), and lower limb function and falls efficacy (5.1%).

**Table 3:** Decomposition of Effects of Variables in Structural Equation Modeling (*N* = 258).

**Variables in the model**	***Estimate***	***S.E*.**	***95% Confidence interval***
Direct effect			
H1 Lower Limb Function on Fatigue	−0.267	0.089	−0.434/−0.071
H2 Falls Efficacy on Fatigue	0.518	0.050	0.415/0.616
H3 Falls Risk on Fatigue	0.559	0.089	0.380/0.731
H4 Falls Risk on Lower Limb Function	−0.277	0.084	−0.427/−0.097
H5 Falls Efficacy on Lower Limb Function	−0.145	0.069	−0.300/−0.020
H6 Falls Risk on Falls Efficacy	0.358	0.094	0.188/0.544
Indirect effect			
Falls Risk on Fatigue through *Lower Limb Function*	0.082	0.039	0.024/0.174
Falls Risk on Fatigue through *Falls Efficacy*	0.205	0.063	0.104/0.344
Falls Risk on Fatigue through *Falls Efficacy and Lower Limb Function*	0.015	0.010	0.003/0.046
Total indirect effect	0.303	0.072	0.173/0.460

## Discussion

As for these hypotheses, fatigue, lower limb function, falls efficacy, and falls risk had significant relationships with each other in our sample. In addition, as far as we know this study is the first to examine the underlying internal mechanism of fatigue on falls risk among the elderly in China. We found that fatigue had direct and indirect effects on falls risk through multiple mediations, while verifying the role of lower limb function and falls efficacy on the chain of mediators that act in this relationship.

To our knowledge, very few studies have assessed the link between fatigue and risk of fall in community-dwelling older subjects. As expected, fatigue was a significant risk factor for falling in older population, as demonstrated by the previous study (39). The direct relationship between fatigue and falls risk we reported has been testified in a systematic review (5). Similarly, recent findings suggested that the severity of fatigue was associated with the risk of falls for community dwelling older adults even after adjustment for possible confounding factors, which is consist with our findings (40). This implies that the importance of fatigue in predicting incidence or risk of falls is applicable to Chinese older adults. Furthermore, among all multiple paths from fatigue to falls risk, mediating the path through falls efficacy was found to be dominant in this study (β = 0.205, *S.E*. = 0.063, *95% CI* 0.104/0.344). Falls efficacy originated from self-efficacy theory (SET) (41), which referred to an individual's perception of capabilities at avoiding falls during essential, nonhazardous activities of daily living (42). In consideration of falls risk in older persons, falls efficacy should not be underestimated. Our result tended to support that the assessment of falls efficacy may be nearly a specific tool to assess falls risk. Meanwhile, there are relatively few studies that directly examine the relationship between fatigue and falls efficacy, our findings also provide evidence that improving fatigue can facilitate falls efficacy in one's personal life, thereby reducing falls risk.

Our research showed through the results of the Bootstrap test program for the intermediary effect that fatigue can affect falls risk among Chinese older adults through lower limb function, which affected through part of the intermediary effect of falls efficacy. In other words, lower limb function partly affected falls risk by influencing falls efficacy. This verified H1 to H5 to h6 that “*lower limb function and falls efficacy”* played a chained intermediary role between fatigue and falls risk, which showed that people with less fatigue are more likely to have better lower limb function, leading to higher falls efficacy, to promote falls risk among the elderly in China. From the perspective of gerontology, the weakness of physiological function made the elderly more susceptible to fatigue, which in turn affects the range of lower limb motion to some extent (20). On the other hand, reduced capacity to produce strength in the lower limbs was an important fall predictor among older adults because the capacity to recover balance depended on the magnitude and rate of joint torques that was produced (43). All of these changes in lower limb function had a significant impact on falls efficacy in older adults. That is to say, older adults with poorer lower limb function have lower falls efficacy (44), meaning less confidence in the activities they engage in. Then, the elderly with low falls efficacy tend to limit their own activities in daily activities (45), which gradually reduces lower limb function, thus entering a vicious cycle, namely “poor lower limb function–reduced falls efficacy–worse lower limb function”. In general, fatigue can affect falls risk through lower limb function and falls efficacy in our sample.

This study also has some limitations. First, due to some objective factors, we adopted a cross-sectional study design, which prevented us from exploring a potential causal relationship. Although existing studies have provided a solid foundation, the current results can be enriched and expanded through further longitudinal studies. Second, although we used a series of questionnaires, we may still miss some potential influencing factors. Future work should incorporate other relevant factors actively to complete a more comprehensive exploration of structural equation modeling. Third, according to the sociodemographic data, our participants had higher education level, more monthly income, and lower medical burden, which may be related to the city in which the study was conducted. The samples for this study were contacted and recruited from an urban community in Beijing, China. As we all know, Beijing is the capital of China, and the elderly who settled here may have superior material conditions to take care of their own health. In the next, we plan to carry out a wider range of survey to improve the generalizability of our findings. Finally, there may be some threats because the independent variables and outcomes are all self-reported. For example, the participants in this study were aged 75 years and above, who were prone to memory loss, so recall bias may occur when filling in the self-report questionnaires. Additionally, when answering research questions, a person may feel the need to answer in a way that conforms to social norms, which could lead to the emergence of social desirability biases. Future work needs to consider the combination of quantitative and qualitative research, so as to better understand how Chinese older adults understand fatigue and falls risk with minimal bias.

## Conclusion

In conclusion, this study investigates how fatigue influences falls risk among the elderly in China. There are many mediating paths between fatigue and falls risk. Falls efficacy and lower limb function were the main mediating variables. Specifically, the results also confirmed a chain mediation model. According to our results, the development and implementation of intervention strategies focusing on lower limb function and falls efficacy may be helpful to reduce the risk of falls for older adults in China. These results may help healthcare professionals to better understand the inherent relationship between fatigue and fall risk that may benefit older adults.

## Data Availability Statement

The raw data supporting the conclusions of this article will be made available by the authors, without undue reservation.

## Ethics Statement

The studies involving human participants were reviewed and approved by Chinese PLA General Hospital Medical Ethics Committee. The patients/participants provided their written informed consent to participate in this study.

## Author Contributions

YH conceived the idea with HP. HP supervised the project. YH drafted the manuscript. HZ revised the manuscript. All authors contributed to collect the data and do the data analysis. All authors contributed to the article and approved the submitted version.

## Funding

This work was supported by the National Key Research and Development Program of China (2018YFC2001400).

## Conflict of Interest

The authors declare that the research was conducted in the absence of any commercial or financial relationships that could be construed as a potential conflict of interest.

## Publisher's Note

All claims expressed in this article are solely those of the authors and do not necessarily represent those of their affiliated organizations, or those of the publisher, the editors and the reviewers. Any product that may be evaluated in this article, or claim that may be made by its manufacturer, is not guaranteed or endorsed by the publisher.
